# PARK(ing) time–How *park* deficiency affects the biological clock in a *Drosophila* model of Parkinson's disease

**DOI:** 10.1002/1873-3468.70389

**Published:** 2026-06-16

**Authors:** Kamila Zientara, Kinga Skoczek, Elzbieta Pyza, Milena Damulewicz

**Affiliations:** ^1^ Department of Cell Biology and Imaging, Institute of Zoology and Biomedical Research, Faculty of Biology Jagiellonian University Krakow Poland

**Keywords:** circadian clock, *Drosophila melanogaster*, oxidative stress, Parkinson's disease

## Abstract

Sleep and circadian disruptions are early symptoms of Parkinson's disease (PD), which is one of the most common neurodegenerative disorders. However, PD has an idiopathic origin, and the factors accelerating the progression of symptoms are not fully understood. One genetic factor associated with PD is a mutation in the parkin gene, which impairs mitophagy and increases oxidative stress. In this research, we used *Drosophila melanogaster* as a model of PD, employing both a *park* ^*1*^ mutant and cell‐specific *park* silencing, and followed the progression of main clock disruption. Our data suggest that pacemaker neurons are sensitive to oxidative stress, and increased ROS levels disrupt daily changes in the morphology of their termini, affecting circadian network communication and sleep regulation.

## Graphical abstract

Drosophila *park* mutants serve as a model for Parkinson's disease. We used this strain to investigate the connection between oxidative stress and the circadian clock mechanism. We showed that increased oxidative stress affects the physiology of pacemaker cells, disrupting their daily structural plasticity. Lack of rhythmic signaling from pacemaker cells has an effect on nighttime sleep time and its fragmentation.

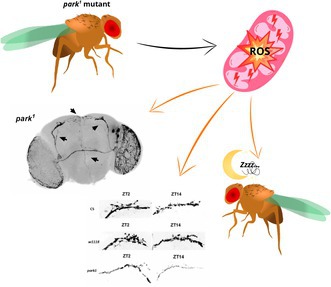

## Abbreviations

**ATG5**, autophagy related gene 5

**BRP**, Bruchpilot

**CRY**, cryptochrome

**CS**, Canton‐S

**DA**, dopaminergic

**hdc**, histidine decarboxylase

**PD**, Parkinson's disease

**PDF**, Pigment Dispersing Factor

**PER**, period

**ple**, pale

**REM**, rapid eye movement sleep

**ROS**, reactive oxygen species

**RpL32**, Ribosomal protein 32

**SCN**, suprachiasmatic nucleus

**sLNv**, small ventral lateral neurons

**SREBP**, Sterol regulatory element‐binding protein

**TIM**, timeless

**w**, white

**αATP**, alpha subunit of sodium–potassium pump

Parkinson's disease (PD) is one of the most common age‐related neurodegenerative disorders; however, its idiopathic etiology makes research more challenging, especially in clinical studies. The most common symptoms include motor disturbances, such as bradykinesia, tremor, postural instability, and rigidity. Although different genetic and environmental factors are involved in disease progression, all of them affect dopaminergic cells, ultimately leading to the severe loss of substantia nigra neurons. Motor symptoms signal that PD is in the late stage, when no treatment is available, except for some medications to alleviate symptoms. Early diagnosis of PD might help to delay the disease progression, which is why many studies focus on defects that appear many years before the clinical diagnosis. Among them are sensory deficits and sleep disorders. Changes in sleep, such as delayed or advanced sleep initiation, insomnia, disorders of REM sleep, disrupted circadian rhythm of sleep and wakefulness, sleep fragmentation, and increased sleepiness during the day, occur in more than 60% of PD patients [1]. Another early PD symptom is disruption of visual contrast sensitivity, the ability to distinguish a visual object from its background [2]. As these symptoms appear 12–14 years before motor disabilities, they can provide an additional early sign for PD diagnosis and offer the time to start therapeutic procedures to prevent neuronal loss. Both of these features are strongly connected with the circadian system.

In mammals, the circadian pacemaker (the central clock) is located in the suprachiasmatic nucleus (SCN) [3]. It is a small region of the brain in the hypothalamus, above the optic chiasm. It generates circadian rhythms, which are entrained by external light stimuli received from the retinal ganglion cells expressing melanopsin through the retinal‐hypothalamic tract. Among many rhythms, the SCN controls cyclic production of melatonin in the pineal gland, which is released during the night and degraded by light [4]. Melatonin feeds back to the SCN cells to decrease their neuronal firing and to promote sleep.

A commonly used model in both chronobiology and neurobiology is provided by the fruit fly, *Drosophila melanogaster*. The circadian clock of *Drosophila* is well described on the cellular and molecular levels. The clock network is based on several groups of neurons in the brain, with the pacemaker located in small ventral lateral neurons (sLNv). This group of 4 neurons exclusively produces the neurotransmitter, Pigment Dispersing Factor (PDF), which is rhythmically released in the dorsal part of the brain [5]. In addition, there is a group of five neurons called large ventral lateral neurons (lLNv), which also produce PDF; however, they innervate a different brain area, the optic lobes. Specificity of anti‐PDF immunostaining provides a possibility of efficiently tracking the daily changes in sLNv terminals' complexity. It has been found that the sLNv axonal termini form highly branched processes at the beginning of the day, while at the beginning of the night, the number of branches is significantly lower [6]. In effect, PDF‐expressing cells change their synaptic partners throughout the day [7]. This rhythm is strongly related to sleep regulation, as one of the output pathways affects sleep‐promoting cells in the mushroom bodies [7].

Our previous studies showed that *park* ^*1*^ mutants, a *Drosophila* model of PD, have a longer period of locomotor activity rhythm [8]. It is an effect of changes in the expression pattern of the main clock genes, *period (per)* and *timeless (tim)* [8]. In addition, the other PD model in *Drosophila*, the *pink* mutant, shows decreased expression of proteins involved in synaptic transmission and changes in synapse morphology [9].

In this research, we used the *park* ^*1*^ mutant or cell‐specific *park* silencing, which disrupts mitophagy, to follow the progression of the main clock disruption.

## Materials and methods

### Flies

Flies were maintained under standard conditions: 12 h of light and 12 h of darkness (LD12:12), at a temperature of 25°C, and fed with standard cornmeal/yeast medium.

The following strains of *Drosophila melanogaster* were used: Canton‐S, *w* ^*1118*^, *park* ^*1*^ (BDSC 34747), *Pdf*‐Gal4/Cy0, *ple*‐Gal4 (kind gift from T. Heino, University of Helsinki), *alrm*‐Gal4 (kind gift from T. Heino), UAS‐*parkRNAi* (BDSC 37509), and UAS‐*catalaseRNAi (*VDRC 6283).

### Locomotor activity and sleep

The locomotor activity system (DAMS, Trikinetics) was used to analyze the activity or sleep of a single fly. This system consists of monitors equipped with infrared light‐emitting diodes and detectors, connected to a computer. Each monitor houses 32 small glass tubes sealed at both ends: one by a food and the other by a foam stopper. Every time the fly crosses the infrared beam in front of the emitter/detector pair, a signal is sent to the computer. For sleep analysis, data from the second day in LD12:12 were monitored every 1 min. Only rhythmic flies that survived until the end of the experiment were taken for further sleep analysis. Data were analyzed by a customized Excel calculator published previously by another group and freely available online [10]. A sleep bout was defined by 5 min of inactivity (where inactivity is defined as no beam crossing during 1 min).

### Immunohistochemistry

Heads of flies were collected at selected time points, then fixed in 4% paraformaldehyde in phosphate buffer saline (PBS; pH 7,4) for 1 h, and washed in PBS. In the next step, brains were manually isolated. Prepared samples were fixed again for half an hour, and washed in PBS and three times in 0.2% PBST (PBS with the addition of 0.2% TritonX100). After that, brains were incubated in 5% normal goat serum (NGS) for 30 min first at room temperature and then incubated with mouse primary antibodies against PDF (1:500, Hybridoma Bank) overnight. Afterward, samples were washed three times in 0.2% PBST and incubated with goat anti‐mouse conjugated with Cy3 secondary antibodies (1:500, Jackson Immuno Research) for 2 h. Finally, brains were washed three times in 0.2% PBST and 10 min in PBS. Then, they were mounted in Vectashield medium (Vector) and examined with a Zeiss Meta 710 Laser Scanning Microscope.

For cryosections, 30 heads were decapitated and fixed in 4% paraformaldehyde for 4 h, then cryoprotected (2 × 10 min in PBS, 1 × 10 min in 12,5% sucrose, overnight in 25% sucrose), mounted in Cryomatrix and frozen in liquid nitrogen. Sections, 20 μm thick, were prepared and immunostained with mouse anti‐Bruchpilot (BRP, nc82, Hybridoma Bank, 1:50) or mouse anti‐alpha subunit of the sodium–potassium pump (aATP, a5, Hybridoma Bank, 1:500) sera overnight, and then with anti‐mouse Cy3‐conjugated antibodies (Sigma, 1:500) for 2 h. After washing, samples were mounted in Vectashield medium and examined with a Zeiss Laser Scanning Microscope.

### Sholl analysis

To visualize axon projections of sLN_v_s, whole‐brain confocal images were used. Pictures were taken using a 40 × objective with an optical zoom of two. Galleries between 7 and 19 images were projected in the *x*–*y* axis to obtain a reconstruction of the full trajectory of those axons. Sholl's method in the ImageJ software was used to quantify the axonal arborization in the dorsal protocerebrum. Concentric rings centered at the point where the first dorsal ramification opens up were drawn on each brain hemisphere. The number of intersections of each projection with a particular ring was counted. The total number of intersections was compared between two time points, ZT2 and Z12 (according to [6]).

### qPCR

Thirty males, 7 days old, were decapitated at ZT1 (ZT is Zeitgeber Time, where ZT0 = light‐on and ZT12 = light‐off) under LD12:12 (12 h of light, 12 h of darkness). Every experiment was repeated six times. Total RNA isolation from whole heads was performed using TriReagent (MRC Inc.) according to the manufacturer's protocol. The cDNA for PCR amplification was prepared from 500 ng total RNA using the High‐Capacity cDNA Reverse Transcription kit (Applied Biosystems). cDNA, diluted 1:5, was used for quantitative PCR. Gene expression was examined using Kapa SYBR Fast (Merck) and 7500Fast Real‐Time PCR System (Applied Biosystems). The following genes were examined: *Ribosomal protein 32* (*RpL32*, Forward: ATGCTAAGCTGTCGCACAAATG, Reverse: GTTCGATCCGTAACCGATGT), *Pigment dispersing factor (Pdf*, Forward: GGCCACTCTCTGTCGCTATC, Reverse: ATTAGCTCCGAGTTGCGCTT*), Sterol regulatory element‐binding protein (Srebp*, Forward: GCTCCAAGAGTGCTTGACTG, Reverse: GAGCAGTCCTAGTTTCGAGC), and *histidine decarboxylase* (*hdc*, Forward: TCAAGCGTGCATTTCATCAG, Reverse: TACACAGATACTTGCCGAGC). Melting curve analysis was assessed for product specificity. Data were calculated using a standard curve and shown as a ratio with the *RpL32* expression level.

## Results

**Fig. 1:**
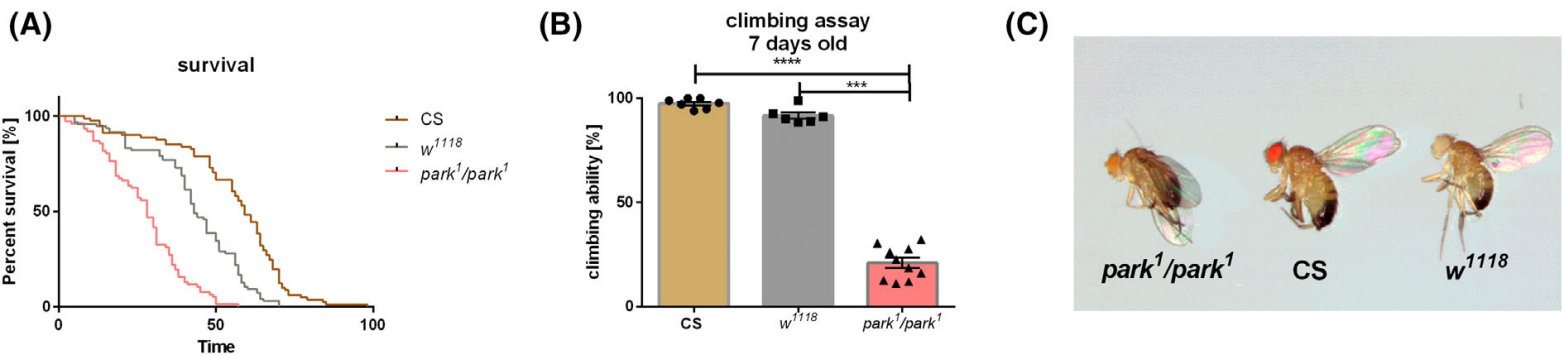
Characteristic changes observed in *park* ^*1*^ mutants. (A) Comparison of survival curves of homozygotic *park* ^*1*^ mutants, wild‐type Canton‐S (CS), and *w* ^*1118*^ flies showed significant changes (Gehan–Breslow–Wilcoxon test, *P* < 0.0001 with both controls), total *N* = 90 for every genotype, 3 replicates. (B) Climbing ability of *park* ^*1*^ mutant flies was decreased (Dunn's test, *park* ^*1*^ vs CS **** *P* < 0.0001, *park* ^1^ vs *w* ^*1118*^ *** *P* = 0.04), total *N* = 90 for every genotype, 10 biologically independent replicates. (C) Picture showing the characteristic wing position of *park* ^*1*^. Bars represent average value ± SEM.

Flies carrying a homozygous mutation of the *park* gene (*park* ^*1*^ */park* ^*1*^) provide one of the genetic PD models. They showed reduced survival (Fig. 1A) [8], impaired climbing ability observed already in young age (Fig. 1B) [11], as well as an atypical posture (Fig. 1C). Because *park* ^*1*^ (strain no. 34747) has a *w*[*] genetic background, we used the additional control, a loss‐of‐function mutation of the *white* (*w* ^*1118*^) gene. The white protein is an ABC‐type guanine transporter, involved in visual pigment transport and aminergic neurotransmitter loading into synaptic vesicles [12], and it has been shown that mutation of this gene affects many aspects of physiology [13]. Mutants of the *park* gene showed shorter lifetime, with their median survival of 28 days, compared with 43 for *w* ^*1118*^ and 59 days for Canton‐S (CS), and maximal lifespan 57 days, while for *w* ^*1118*^ it was 70 days and 98 days for CS (Fig. 1A). In young, 7 days old flies, mortality was similar to the controls; however, their climbing ability was only 20.7% compared with 90.6% for *w* ^*1118*^ and 97.8% for CS (Fig. 1B). *park* ^*1*^ mutants also showed the abnormal posture, with a very characteristic wing position (Fig. 1C).

**Fig. 2:**
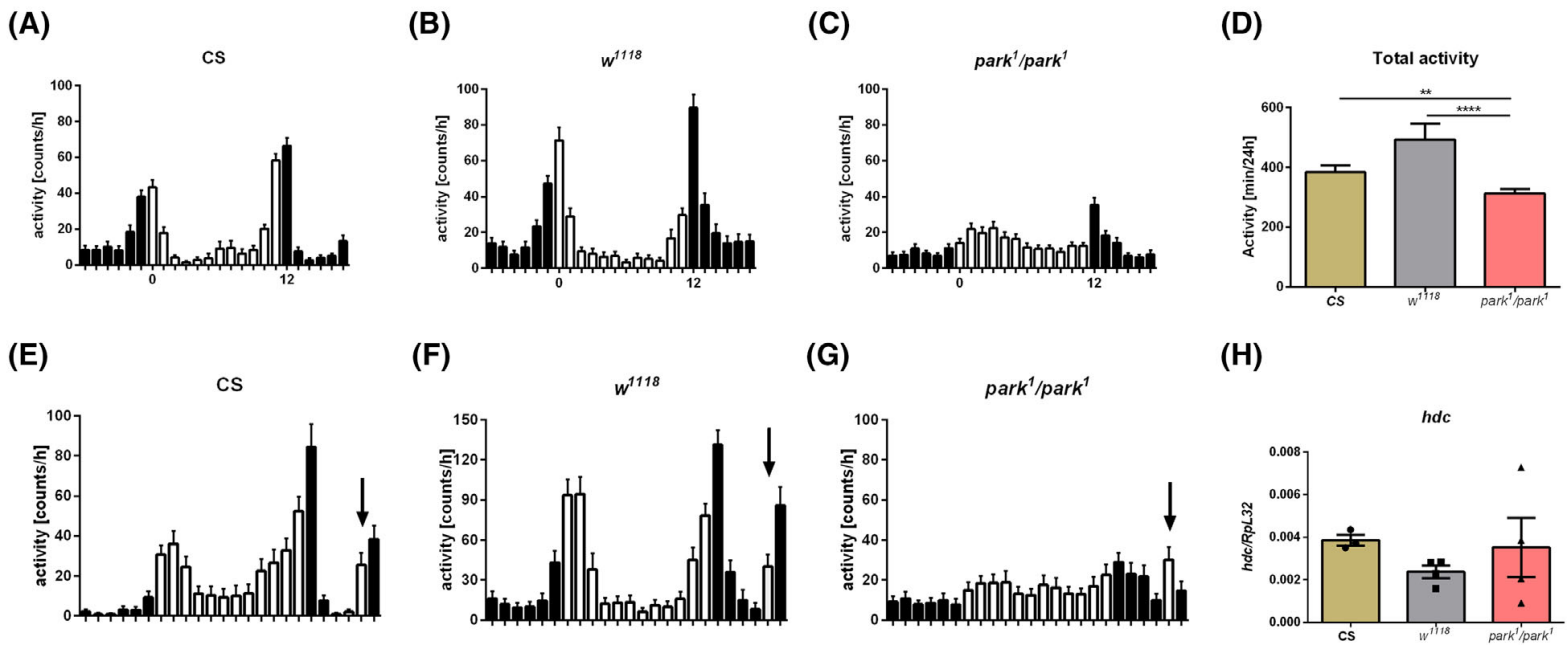
Parkinson's disease (PD) model flies exhibit decreased morning activity, but their response to light is unaffected. (A–C) Daily activity profile of wild‐type Canton‐S (A), *w* ^*1118*^ (B), and *park* ^*1*^ mutant (C) during LD12:12 conditions. ZTs indicate Zeitgeber Time, where ZT0 is the time when the lights are on, and ZT12 is the time when the lights are off. (D) Total activity (minutes) during 24 h showed a significant decrease in *park* ^*1*^ mutant flies (*N* = 85, *t*‐test, *park* ^*1*^ vs CS ** *P* = 0.0081, *park* ^*1*^ vs *w* ^*1118*^ **** *P* < 0.0001). (E–G) Startle effect shown as a daily activity profile of flies with an additional 1‐h light pulse during the night at ZT16 (marked with arrow) (*N* = 96). (H) Gene expression level of *histidine decarboxylase* (*hdc*) is not changed in *park* ^*1*^ mutants compared with controls (*N* = 4). Bars represent average value ± SEM. Every experiment was repeated three times.

The daily activity of *park* mutants under LD12:12 conditions was also changed compared with controls. Wild‐type flies, CS, and *w* ^*1118*^ showed a normal, bimodal pattern of activity with two peaks, at the beginning of the day and at the beginning of the night (Fig. 2A,B). They also showed morning and evening anticipation. The PD model flies did not show the morning peak of activity and also had slightly higher activity at the beginning of the night (Fig. 2C). They also did not show clear morning and evening anticipation, which indicates that their circadian clock is not working properly. Total activity during 24 h was decreased compared with both controls (Fig. 2D).

To check whether the lack of morning peak of activity observed in *park* mutants is connected to a problem with sensitivity to light, flies were exposed to a 1‐hour light pulse during the night. Mutants were sensitive to direct exposure to light during the day and in the middle of the night (startle response), similar to control flies (Fig. 2E–G). However, in CS and *w* ^*1118*^ flies, the effect was still observed 1 hour after light‐off, while *park* ^*1*^ activity was enhanced only after stimulation. In addition, the expression of the *histidine decarboxylase* (*hdc*) gene was not changed (Fig. 2H), suggesting that neurotransmission from photoreceptors is effective in the PD model flies.

**Fig. 3:**
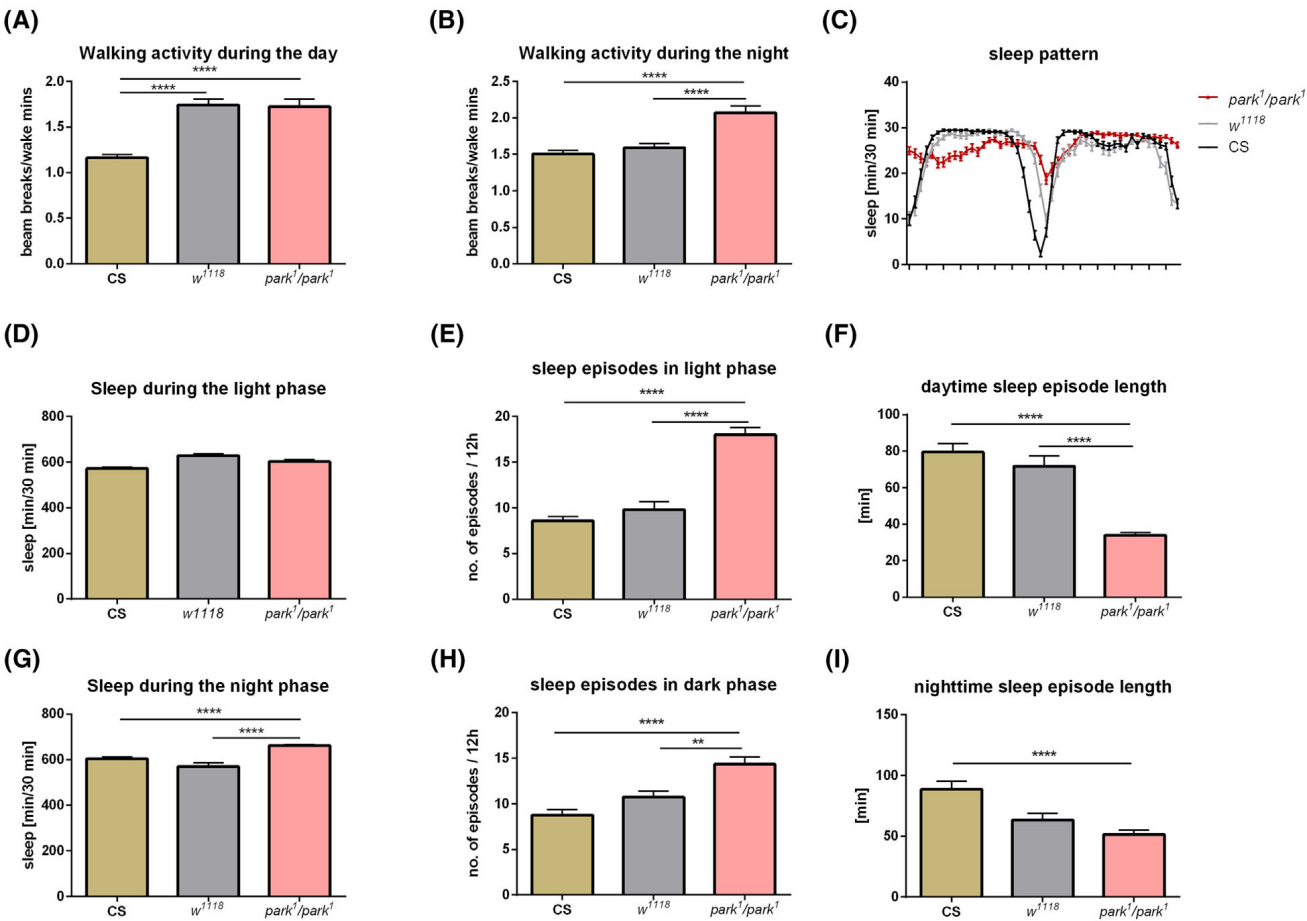
Quality of sleep is affected in *park* ^*1*^ mutants. (A, B) Walking activity in *park* ^*1*^ mutants was increased during the day (**** *P* < 0.0001 with both controls), and during the night, there was a difference only with CS (**** *P* < 0.0001). (C) Daily sleep pattern of Parkinson's disease (PD) model flies showed decreased sleep in the middle of the day, between ZT3 and ZT9 (*N* = 86). (D) Sleep time during the day was similar to controls. (E, F) The number of sleep episodes counted during the day was increased but the length of episodes was decreased (**** *P* < 0.0001 with both controls). (G) sleep time during the night was increased compared with both controls (**** *P* < 0.0001 with both controls), similar to the number of sleep episodes (H, ** *P* < 0.01). (I) Sleep episode length during the night was decreased compared with CS but not with *w* ^*1118*^. Bars represent average value ± SEM. Every experiment was repeated three times.

Although the total activity of mutant flies was decreased compared with controls, their mobility was not limited; they showed even higher walking activity than Canton‐S during the day and night (Fig. 3A,B).

**Fig. 4:**
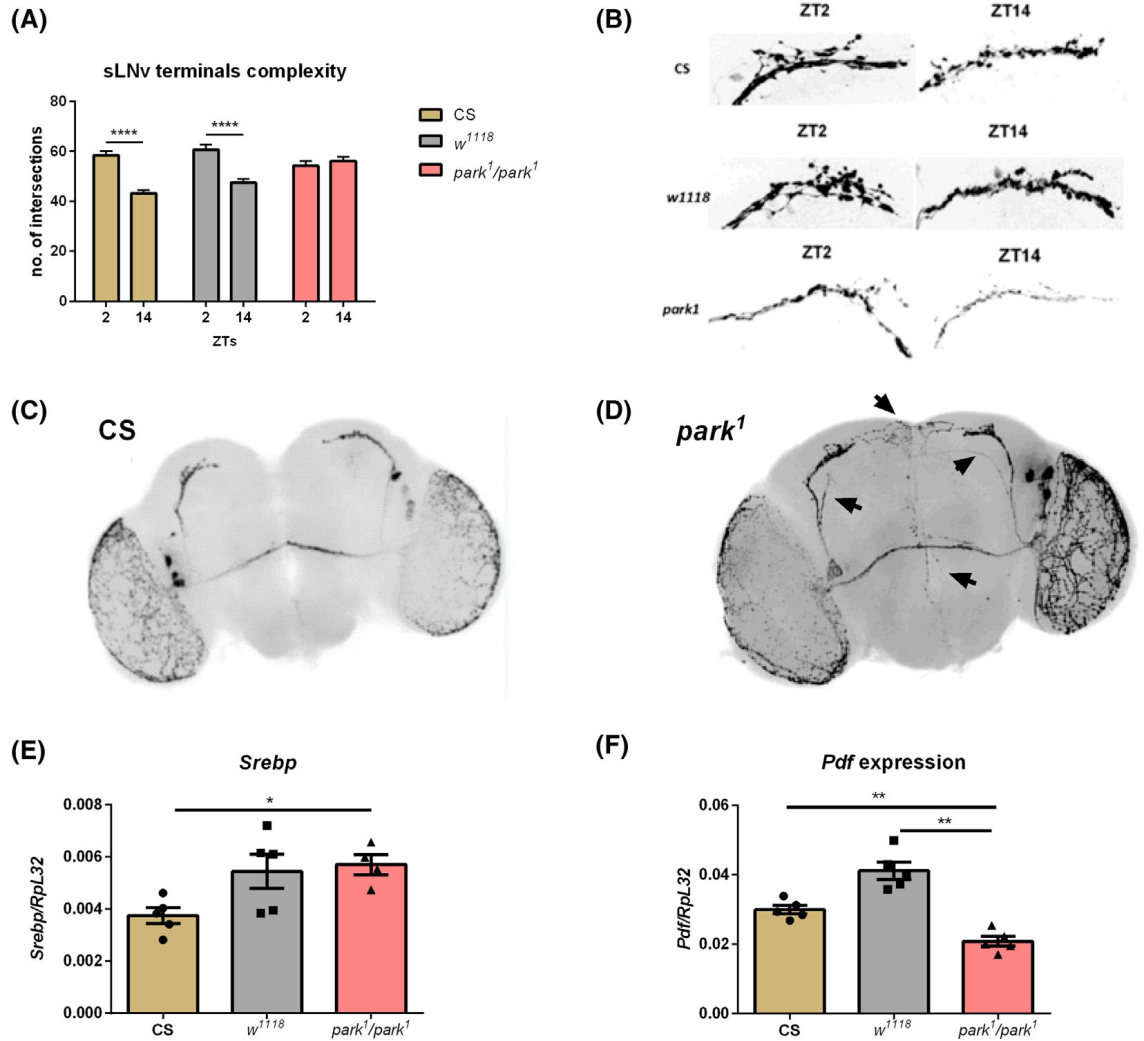
*park* mutation affects daily changes in clock neurons' morphology. (A, B) sLNv terminals complexity measured at the beginning of the day (ZT2) and night (ZT14) is abolished in *park* ^*1*^ mutants (*t*‐test, *P* = 0.4702, *N* = 50–64), while normal in controls with a higher number of intersections at ZT2 than at ZT14 (**** *P* < 0.0001) (CS *N* = 45–63, *w* ^*1118*^ *N* = 54–61). (C, D) *park* mutants show abnormal processes and arborizations of PDF‐expressing cells (marked with arrows). ZT—Zeitgeber Time. (E) Expression of *Sterol regulatory element‐binding protein* (*Srebp*) is higher compared with CS (*N* = 5, Mann–Whitney, * *P* = 0.0156), but similar to *w* ^*1118*^. (F) *Pigment dispersing factor* (*Pdf*) gene expression is decreased compared with CS (*N* = 5, Mann–Whitney, ** *P* = 0.0079) and *w* ^*1118*^ (** *P* = 0.0079). Bars represent average value ± SEM. Every experiment was repeated at least three times.

The *park* mutants displayed also severe changes in sleep architecture, especially during siesta time (Fig. 3C). Although total sleep time during the day was unchanged (Fig. 3D), flies showed more sleep episodes during the light phase (Fig. 3E), and the average time of the episode was shorter than in control flies (Fig. 3F). Night sleep was strongly affected with longer total time of sleep (Fig. 3G), with more frequent (Fig. 3H) and shorter sleep episodes (Fig. 3I). The observed sleep changes seem to be connected to the clock disruption, which is also observed in PD patients and model organisms. In *Drosophila*, one of the important parameters of proper clock function is the daily rhythm in the complexity of sLNv terminals in the dorsal brain. We observed that both control strains showed normal daily patterns of sLNv plasticity, with a higher number of processes at the beginning of the day, and a lower number at the beginning of the night (Fig. 4A,B), similar to what was observed by other authors [6]. However, *park* mutants did not show any daily changes in the structure of sLNv termini (Fig. 4A,B). Surprisingly, 11.7% of *park* mutants showed abnormal axonal projections of PDF‐immunostained neurons (Fig. 4C,D, Table 1). The sLNv processes formed additional contralateral projections and terminated in the opposite hemisphere. In addition, the posterior optic tract (POT), which connects and coordinates lLNvs from two hemispheres, was also more branched than in controls (Fig. 4D). This abnormal morphology suggests that disrupted mitophagy in *park* ^1^ mutants may affect the development of clock neurons. Moreover, *park* mutants showed decreased expression of *Srebp*, encoding sterol regulatory element‐binding protein (SREBP) (Fig. 4E), which indicates disrupted lipid metabolism, a process necessary for membrane remodeling.

**Table 1:** Locomotor activity of flies.

Genotype	Period of locomotor activity	% arrhythmic flies	Total *N*	% of abnormal PDF‐immunopositive processes
CS	24.1 ± 0.5	4.3	69	0
*white*	23.7 ± 0.8	3.4	58	0
*park* ^*1*^ */park* ^*1*^	27.2 ± 1.0	26	71	11.7
*ple>parkRNAi*	23.7 ± 0.7	24	79	8.1
*Pdf>parkRNAi*	24.1 ± 0.4	16	66	1.6
*ple>white*	23.7 ± 0.6	8.9	78	0
*white>parkRNAi*	23.6 ± 0.4	11.5	52	0
*Pdf‐Gal4/+*	24.19 ± 0.5	3.6	55	0

In addition to anatomical abnormalities, we also observed lower fluorescence intensity correlated with PDF level in *park* mutants (Fig. 4B). This result was confirmed by a lower level of the *Pdf* gene expression. *Pdf* mRNA level in *park* mutants was significantly decreased compared with that in controls (Fig. 4F). Surprisingly, *Pdf* expression level was the highest in *w* ^*1118*^ flies (Fig. 4F).

**Fig. 5:**
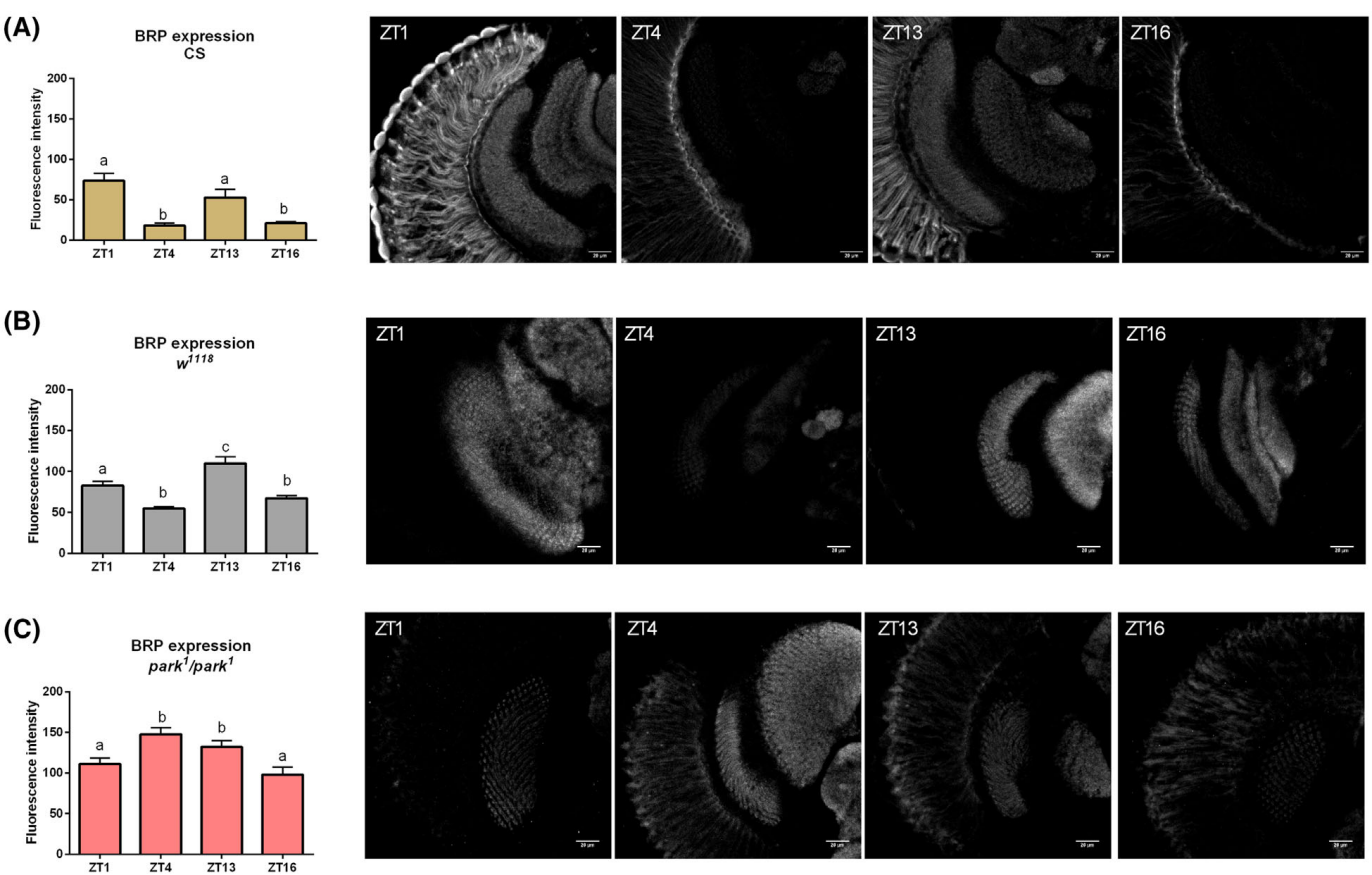
*park* ^*1*^ mutation affects synaptic plasticity in photoreceptor terminals. Daily changes in the expression level of presynaptic protein Bruchpilot (BRP) in the tetrad synapses of the retina photoreceptors in the lamina. (A, B) Control flies show the strongest anti‐BRP immunosignal at the beginning of the day (ZT1) and night (ZT13) (CS *N* = 19–21, *w* ^*1118*^ *N* = 58–60, Tukey's test). (C) *park* ^*1*^ mutants show the highest BRP expression at ZT4 and ZT13 (*N* = 94, Dunn's test). ZT—Zeitgeber Time. Statistically significant differences are marked as different letters above the bars, and detailed statistics are presented in Table S1. Bars represent average value ± SEM. Every experiment was repeated three times. Scale bars = 20 μm.

Light is required to entrain the pacemaker in long‐term experiments. One of the key mechanisms of rhythmic signaling from photoreceptors to the deeper parts of the brain is the daily rhythmicity of the presynaptic protein Bruchpilot levels in the R1‐R6 photoreceptor terminals. In wild‐type flies, BRP in the distal lamina, the first neuropil of the optic lobe, shows two peaks in abundance, 1 hour after light‐on (ZT1) and 1 hour after light‐off (ZT13) (Fig. 5A) [14]. This was observed in both controls, Canton‐S (Fig. 5A) and *w* ^*1118*^ (Fig. 5B). However, in *park* mutants, this pattern was changed, with higher BRP expression observed from the middle of the day to the beginning of the night (Fig. 5C).

**Fig. 6:**
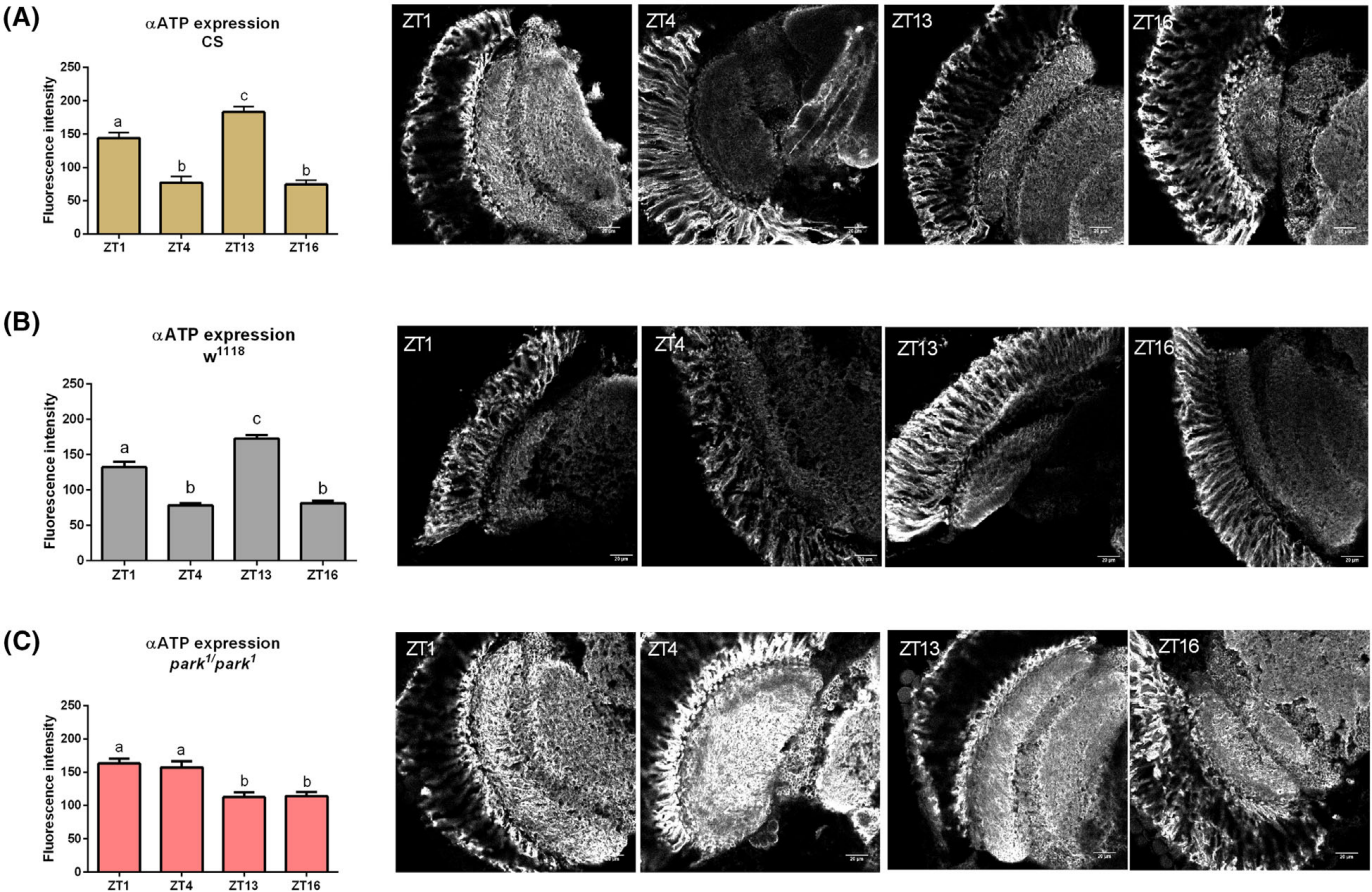
*park* ^*1*^ mutation affects daily changes in glia activity. The alpha subunit of the sodium–potassium pump (α‐ATPase) shows daily changes in expression in epithelial glia in the lamina. (A, B) Control flies show the strongest immunosignal at the beginning of the day (ZT1) and night (ZT13) (CS *N* = 22–38, *w* ^*1118*^ *N* = 56–60, Tukey's test). (C) *park* ^*1*^ mutants show the highest BRP expression during the day, at ZT1 and ZT4 (*N* = 88–92, Dunn's test). ZT—Zeitgeber Time. Statistically significant differences are marked as different letters above the bars, and detailed statistics are presented in Table S1. Bars represent average value ± SEM. Every experiment was repeated three times. Scale bars = 20 μm.

Synaptic transmission from the eye photoreceptors to the first‐order interneurons is modulated by the lamina epithelial glial cells [15, 16]. The activity of glial cells can be measured by expression of the alpha subunit of the sodium–potassium ATPase [17]. In wild‐type flies and *w* ^*1118*^, the level of ATPase alpha subunit showed a bimodal pattern, with the maxima at the beginning of the day and at the beginning of the night (Fig. 6A,B) [17, 18]. However, in *park* mutants, the ATPase alpha subunit level in the lamina was higher during the day and lower during the night (Fig. 6C).

**Fig. 7:**
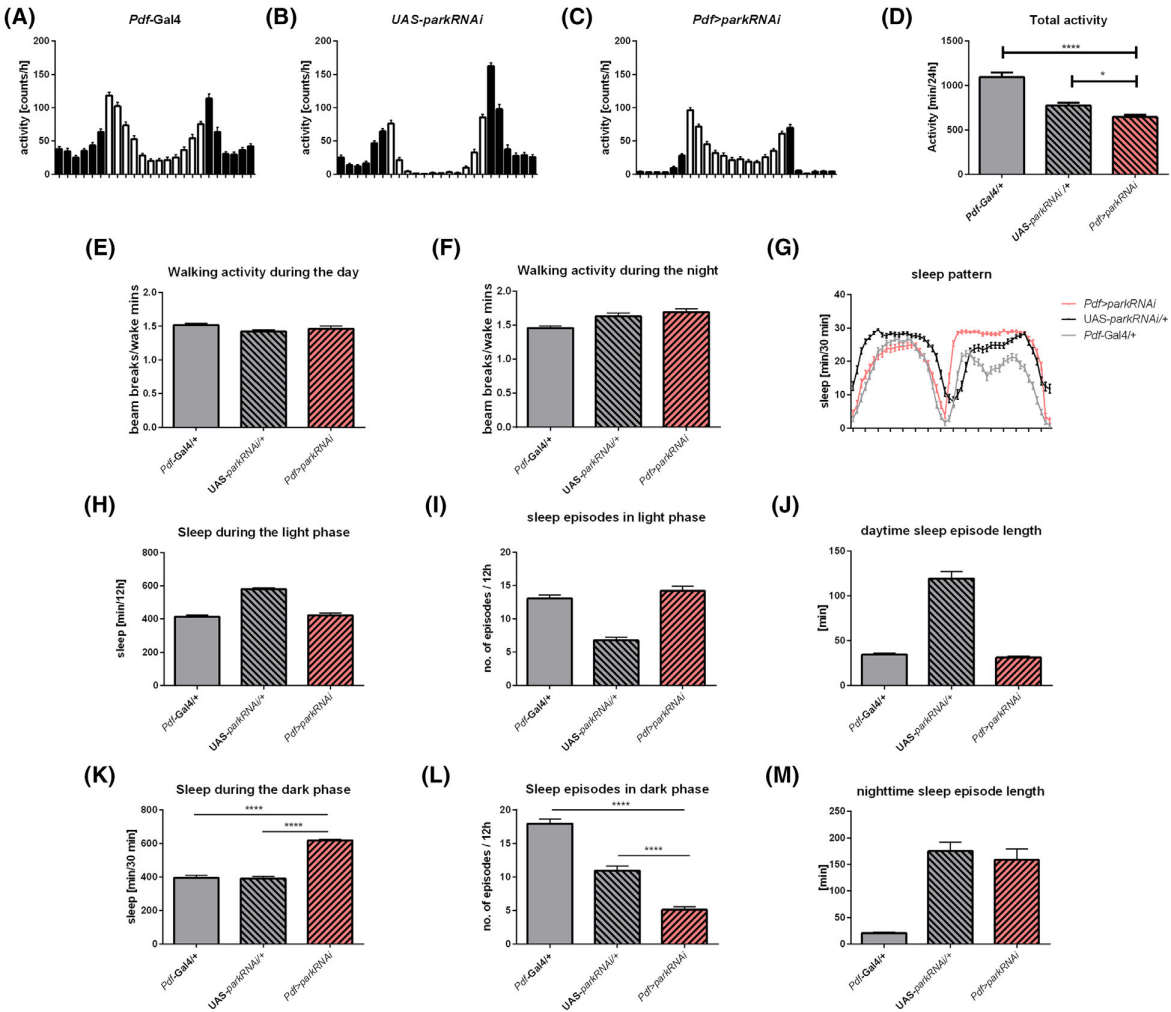
Mitophagy disruption in clock neurons affects nighttime sleep. Activity and sleep profile of flies with *park* silencing specifically in LNv (*Pdf>parkRNAi*). (A–C) Daily activity profile of parental (A, B) and experimental strains (C). (D) Total activity during 24 h is decreased in experimental flies compared with both controls (Bonferroni's test, * *P* = 0.0183 with UAS‐*parkRNAi*, **** *P* < 0.0001 with *Pdf‐*Gal4). (E, F) walking activity is not affected. (G) Daily sleep profile shows increased sleep during the night. (H–J) Sleep time, the number of sleep episodes, and average length of sleep bout during the day are not changed compared with both controls. (K) Sleep time is increased during the night (Bonferroni's test, **** *P* < 0.0001 with both controls). (L) The number of sleep episodes is lower during the night in *Pdf>parkRNAi* flies (Bonferroni's test, **** *P* < 0.0001 with both controls). (M) the average length of sleep episode is not affected. Total number of individuals: *Pdf*‐Gal4 = 52, UAS‐*parkRNAi* = 63, *Pdf>parkRNAi* = 66. Bars represent average value ± SEM. Every experiment was repeated three times.

The effect of PARK on the clock neurons is difficult to examine with mutants, as the lack of this protein affects every cell in the body. To investigate which cells involved in circadian regulation are the most affected by mitophagy disruption, we silenced *park* in clock neurons (using *Pdf*‐Gal4), astrocytes (*alrm*‐Gal4), and dopaminergic neurons (*ple*‐Gal4), respectively. Control flies, *Pdf*‐Gal4/+ and UAS‐*parkRNAi*/+ showed the normal daily pattern of activity with morning and evening peaks (Fig. 7A,B); however, UAS‐*parkRNAi*/+ flies were not active during the day between ZT3 and ZT9 (Fig. 7B). Interestingly, *Pdf>parkRNAi* flies were inactive during the night, except for the transitions between the day and night (ZT12) and the night and day (morning anticipation at ZT23) (Fig. 7C). Total activity of experimental flies was also decreased (Fig. 7D), even though the walking activity was not changed (Fig. 7E,F). Siesta time was not affected, with normal sleep time, pattern, and sleep episode number compared with control flies (Fig. 7G–J). Surprisingly, although the sleep time was increased during the whole night (Fig. 7G–K), the number of sleep episodes was lower during the night with the normal average length compared with both controls (Fig. 7L,M), which means that the effect of *park* silencing was different than sleep changes observed in the *park* mutant.

**Fig. 8:**
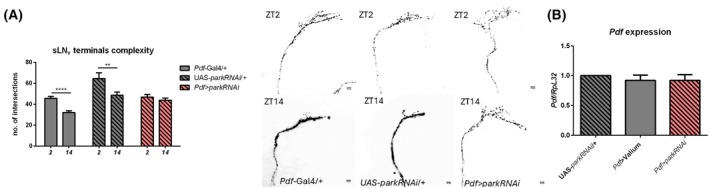
Mitophagy disruption in clock neurons affects daily plasticity of sLNv in the PDF‐independent pathway. (A) Daily remodeling of sLNv processes of parental flies shows daily changes (*t*‐test, *Pdf*‐Gal4 **** *P* < 0.0001, UAS‐parkRNAi ** P = 0.0085), while in *Pdf>parkRNAi* flies, it is not maintained (*P* = 0.5385). Scale bars = 10 μm. Total number of brains analyzed: *Pdf*‐Gal4 = 32–37, UAS‐*parkRNAi* = 21–26, *Pdf>parkRNAi* = 44–47. ZT—Zeitgeber Time. (B) The expression level of the *Pdf* gene is not changed after *park* silencing in LNv neurons. Bars represent average value ± SEM. Every experiment was repeated three times.

Decreased level of PARK in the pacemaker cells also disrupted neuronal plasticity of PDF‐expressing cells (Fig. 8A); however, abnormal arborization was observed only in 1.3% of the examined brains (Table 1), and the level of *Pdf* mRNA was not affected (Fig. 8B).

**Fig. 9:**
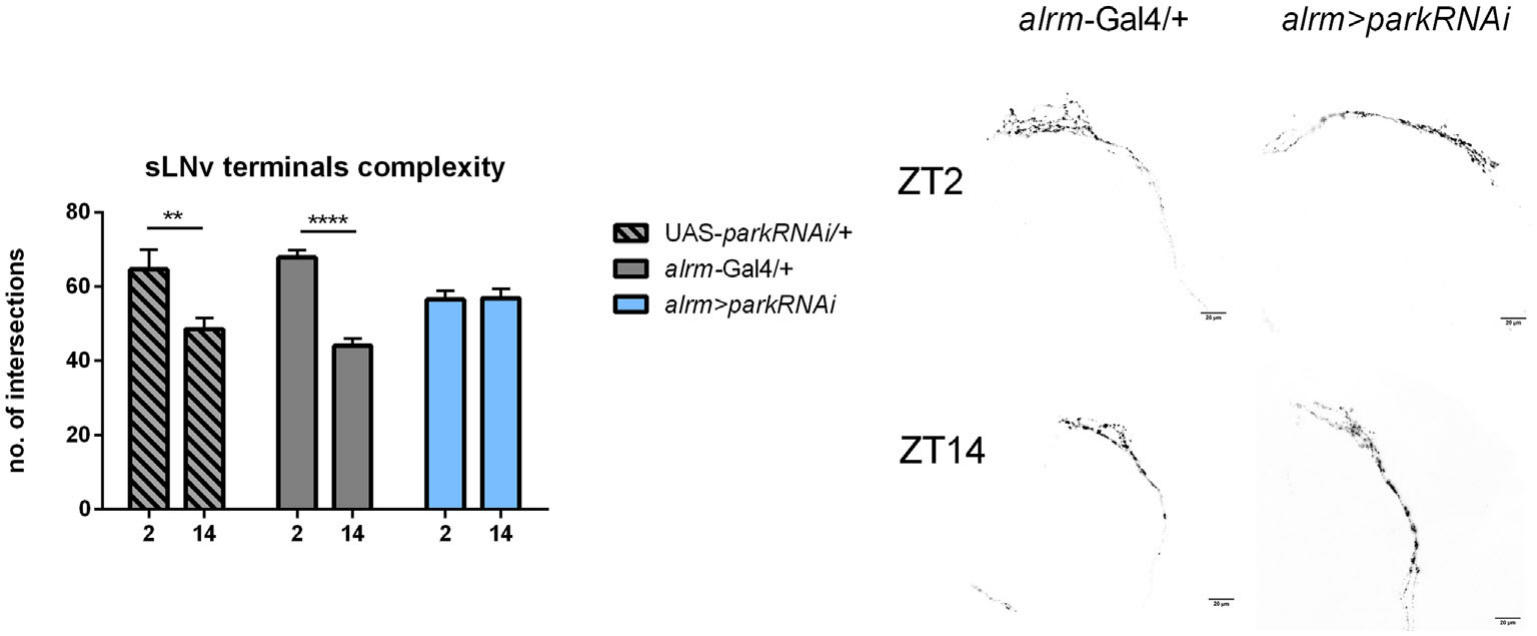
PARK in astrocytes affects daily changes in the structural plasticity of sLNv terminals. Daily remodeling of sLNv processes of parental flies shows daily changes (UAS‐*parkRNAi* ** *P* < 0.01, *alrm*‐Gal4 **** *P* < 0.0001, *N* = 42–50) while in *alrm>parkRNAi* flies it is not maintained (*P* > 0.9999, *N* = 39–51). ZT—Zeitgeber Time. Bars represent average value ± SEM. Every experiment was repeated three times. Scale bars = 20 μm.

**Fig. 10:**
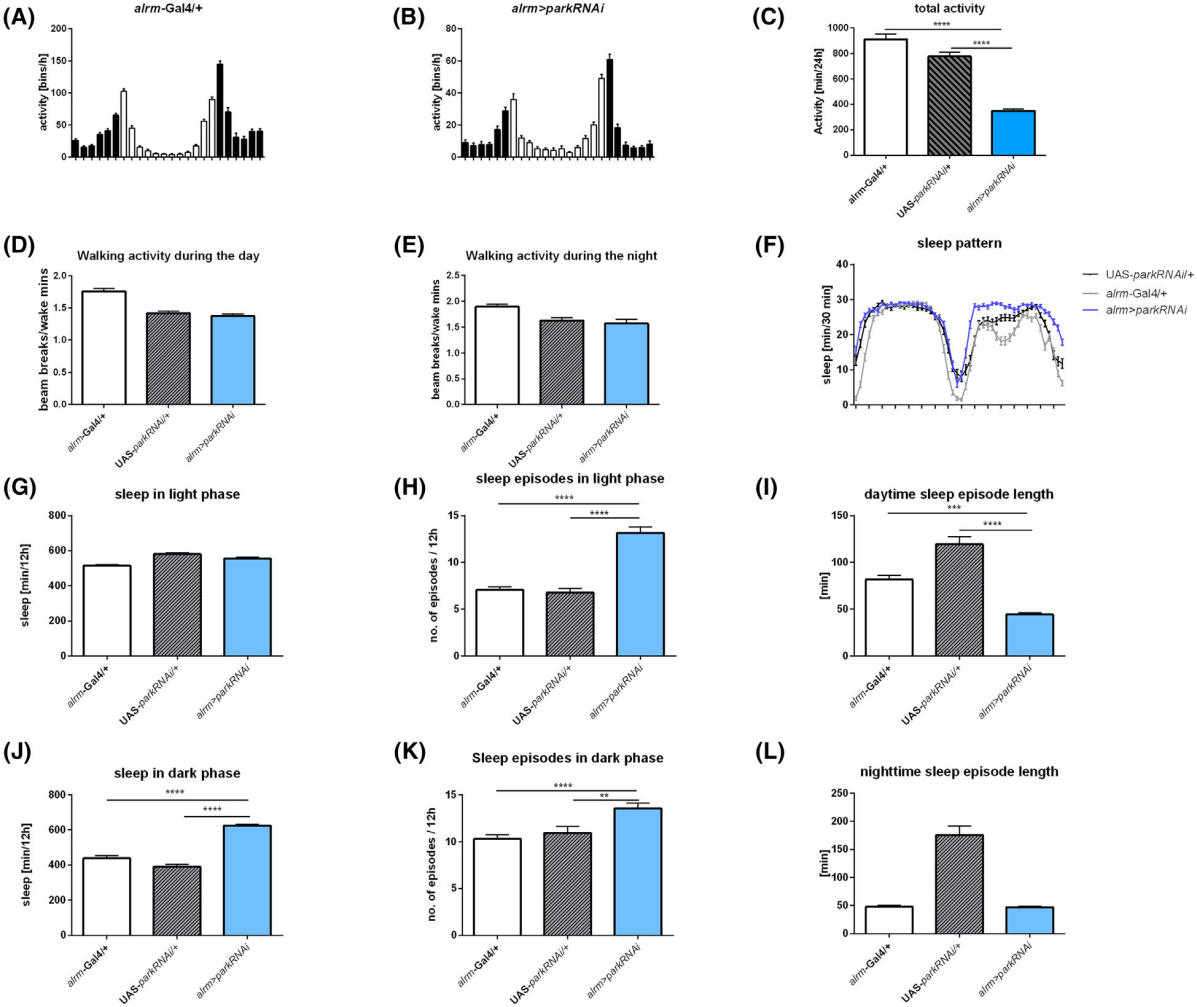
*park* silencing in astrocytes (*alrm>parkRNAi*) affects sleep length and quality. (A, B) Daily activity profile of control (*alrm*‐Gal4) and experimental flies. (C–E) Total activity of experimental flies is decreased compared with both controls (Bonferroni's test, **** *P* < 0.0001), but walking activity is not affected. (F) Daily pattern of sleep shows increased sleep time during the night (ZT13‐ZT24). (G–I) Sleep time during the day (siesta) is not changed; however, it is composed of a higher number of shorter episodes (H: Bonferroni's test, **** *P* < 0.0001; I: **** *P* < 0.0001, *** *P* = 0.0005 with *alrm*‐Gal4). (J–L) Sleep time during the night is longer compared with both controls (Bonferroni's test, **** *P* < 0.0001 with both controls), with an increased number of sleep episodes (Bonferroni's, **** *P* < 0.0001 with *alrm*‐Gal4 and ** *P* = 0.0029 with UAS‐*parkRNAi*), but the average length of sleep bout is similar to that of the parental group. Total number of individuals *alrm*‐Gal4 = 95, UAS‐*parkRNAi* = 63, *alrm>parkRNAi* = 94. Bars represent average value ± SEM. Every experiment was repeated three times.

To check the effect of decreased PARK level on sLNv daily membrane remodeling, *park* expression was silenced in cells that communicate with clock neurons, such as astrocytes and dopaminergic neurons. Flies with expression of *parkRNAi* specifically in astrocyte‐like glia (*alrm* driver) showed no daily rhythm in sLNv terminal branching (Fig. 9). Although locomotor activity pattern was normal (Fig. 10A,B), total activity was strongly decreased (Fig. 10C), with no change in walking activity (Fig. 10D,E). Analysis of the sleep architecture (Fig. 10F,G) and parameters showed that even though the time of sleep during the day was not changed, its quality was decreased with too many short sleep episodes (Fig. 10H,I). Nighttime sleep was longer (Fig. 10J) with an increased number of bouts (Fig. 10K), but the average length of the episode was not changed (Fig. 10L).

**Fig. 11:**
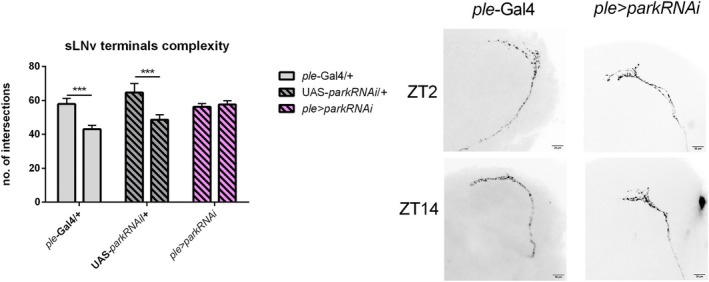
Structural plasticity of sLNv terminals depends on the condition of dopaminergic cells. Daily remodeling of sLNv processes of parental flies shows daily changes (*t*‐test; *** *P* < 0.001), while in flies with lower expression of PARK in dopaminergic cells (*ple>parkRNAi*), this rhythmicity is not observed (*P* = 0.7309). ZT—Zeitgeber Time. The number of brains analyzed: *ple*‐Gal4 = 28–29, UAS‐*parkRNAi* = 21–26, *ple>parkRNAi* = 63. Bars represent average value ± SEM. Every experiment was repeated three times. Scale bars = 20 μm.

**Fig. 12:**
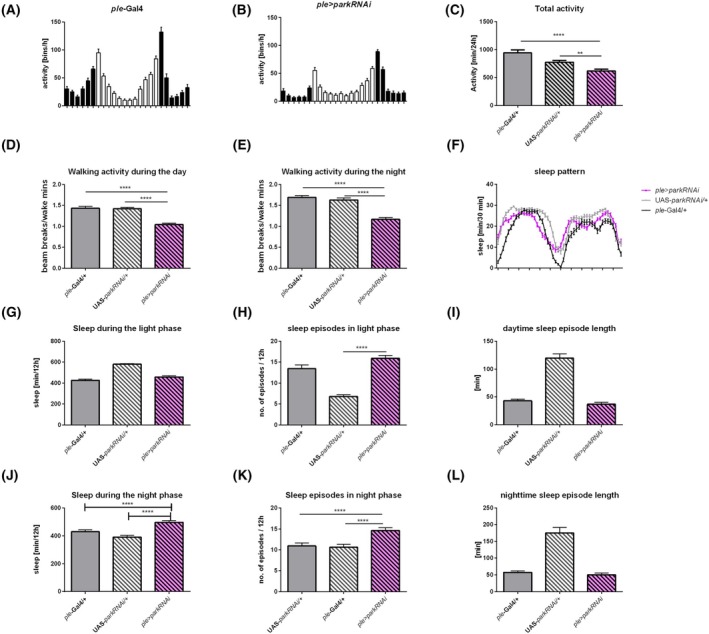
Mitophagy disruption in dopaminergic cells (*ple>parkRNAi*) affects sleep time during the night but does not change the siesta. (A, B) Daily activity profile of control (*ple*‐Gal4) and experimental flies. (C–E) Total activity of experimental flies is lower than in both controls (Bonferroni's test, **** *P* < 0.0001 with *ple*‐Gal4, ** *P* = 0.0054 with UAS‐*parkRNAi*), and walking activity is also decreased both during the day and night (Bonferroni's test, **** *P* < 0.0001). (F) Daily pattern of sleep shows decreased sleep time at the end of the day. (G–I) Sleep time and quality during the day are not changed in *ple>parkRNAi* flies. (J) Sleep time during the night is longer compared with both controls (Bonferroni's test, **** *P* < 0.0001). (K, L) The number of sleep episodes is increased (Bonferroni's test, **** *P* < 0.0001), but the average length of sleep bout is similar to that of at least one control group. The number of individuals: *ple*‐Gal4 *N* = 53, UAS‐*parkRNAi N* = 63, *ple>parkRNAi N* = 79. Bars represent average value ± SEM. Every experiment was repeated three times.

Finally, the decrease of PARK in dopamine‐producing cells *(ple>parkRNAi*) also disrupted the daily sLNv terminal plasticity (Fig. 11). The locomotor activity profile of these flies was maintained (Fig. 12A,B); however, morning and evening peaks showed lower amplitude, and total activity time was decreased compared with both control strains (Fig. 12C), while walking activity was increased during the night (Fig. 12D,E). Sleep profile of *ple>parkRNAi* flies was only slightly changed at the end of the day (Fig. 12F), the amount of sleep during the day was similar to control (Fig. 12G), with a higher number of episodes (Fig. 12H,I). During the dark phase, flies slept longer (Fig. 12J) but in more frequent intervals (Fig. 12K,L).

**Fig. 13:**
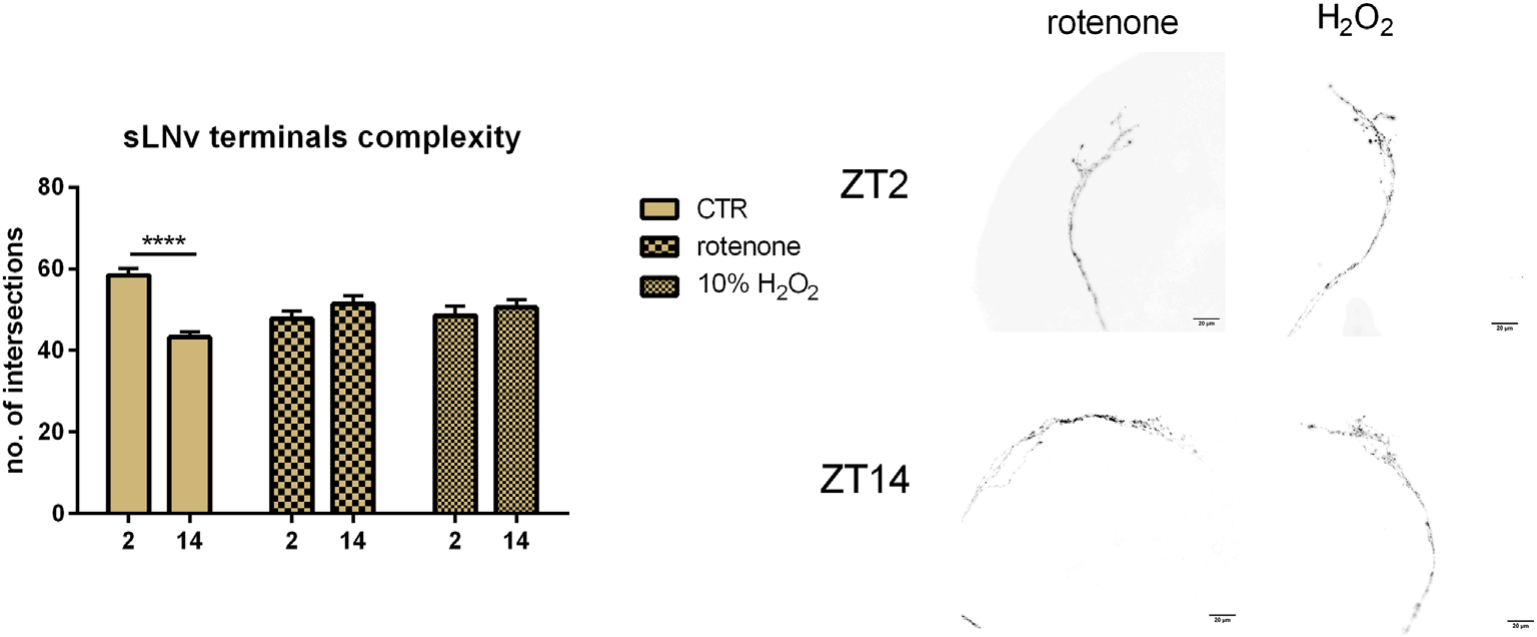
Oxidative stress disrupts the daily plasticity of clock neurons' terminals. Feeding with rotenone for 2 days or 10% H_2_O_2_ for 4 h affects daily remodeling of sLNv processes. ZT—Zeitgeber Time. The number of brains analyzed: CTR *N* = 45–63, rotenone *N* = 46–54, H_2_O_2_ *N* = 27–29. Sidak's test: CTR **** *P* < 0.0001, rotenone *P* = 0.3718, H_2_O_2_ *P* = 0.9188. Bars represent average value ± SEM. Every experiment was repeated three times. Scale bars = 20 μm.

**Fig. 14:**
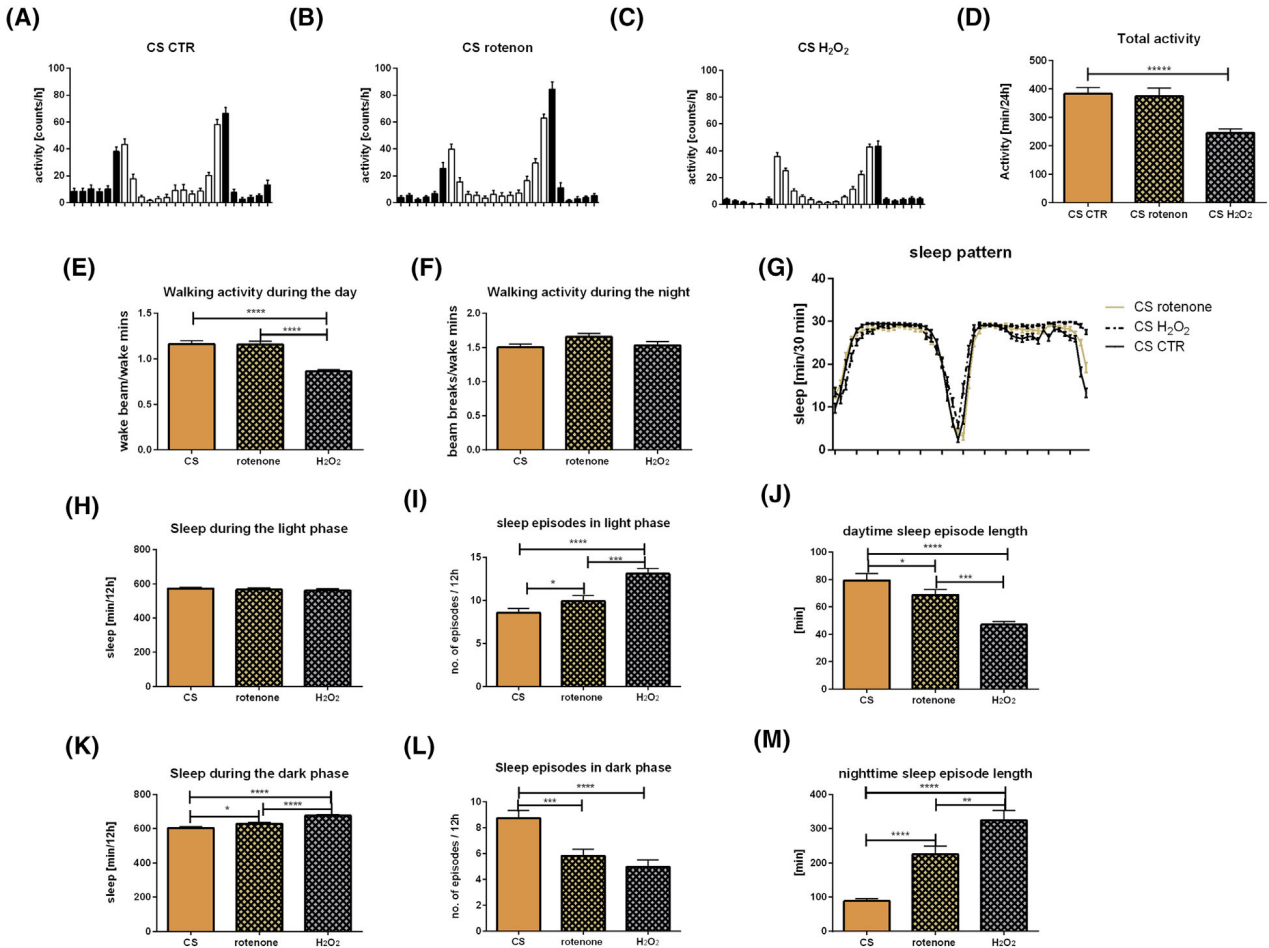
Oxidative stress affects sleep. Activity and sleep analysis of flies fed with rotenone or H_2_O_2_. (A–C) Daily activity profiles of control (CS CTR) and experimental flies. (D) Total activity of experimental flies is decreased after H_2_O_2_ exposure (Bonferroni's test, **** *P* < 0.0001). (E) Walking activity during the day is decreased after H_2_O_2_ treatment (Bonferroni's test, **** *P* < 0.0001), but not after rotenone feeding. (F) Walking activity during the night is not affected. (G) Daily pattern of sleep shows increased sleep during the night after H_2_O_2_ feeding. (H, I) Sleep time during the day is not affected, but the number of sleep episodes is higher (Bonferroni's test, **** *P* < 0.0001 CS vs H_2_O_2,_ * *P* = 0.0098 CS vs rotenone, *** *P* < 0.001). (J) the average sleep episode length is decreased (Bonferroni's test, CS vs H_2_O_2_ **** *P* < 0.0001, * *P* = 0.0093 CS vs rotenone, *** *P* < 0.001) (K) Sleep time during the night is increased compared with control flies (rotenone * *P* = 0.0073 and H_2_O_2_ **** *P* < 0.0001). (L) Total number of sleep episodes during the night is decreased under oxidative stress conditions (**** *P* < 0.0001, *** *P* < 0.001), M: with longer bouts (**** *P* < 0.0001, ** *P* < 0.01). Total number of flies: CTR = 66, rotenone = 80, H_2_O_2_ = 74. Bars represent average value ± SEM. Every experiment was repeated three times.

The daily neuronal plasticity of sLNv terminals was not maintained, no matter which cells had decreased PARK level, which indicates that increased cellular stress could affect this rhythmic plasticity. To check this possibility, flies were fed with the neurotoxin rotenone for 2 days or with 10% H_2_O_2_ for 4 h. Rotenone exposure increases dopaminergic cells' neurodegeneration; however, this effect is observed after 7 days of the treatment. After a short‐term exposure, the oxidative stress is highly increased; however, cells are not in an apoptotic state yet [19]. Indeed, flies fed with rotenone for 2 days did not show changes in the sLNv terminal complexity (Fig. 13). Similarly, flies fed with H_2_O_2_ did not show changes in the sLNv terminal complexity between selected time points (Fig. 13). They also showed the normal profile of daily locomotor activity (Fig. 14A–C).

Flies treated with H_2_O_2_ showed more severe changes with decreased locomotor activity (Fig. 14D) followed by lower walking activity during the day (Fig. 14E). Sleep profile and the level during the day were not changed (Fig. 14G,H), more sleep episodes with decreased bout duration were observed during the light phase (Fig. 14I, J). During the night, flies spend less time inactive, with a decreased number of longer sleep episodes (Fig. 14F, K–M).

**Fig. 15:**
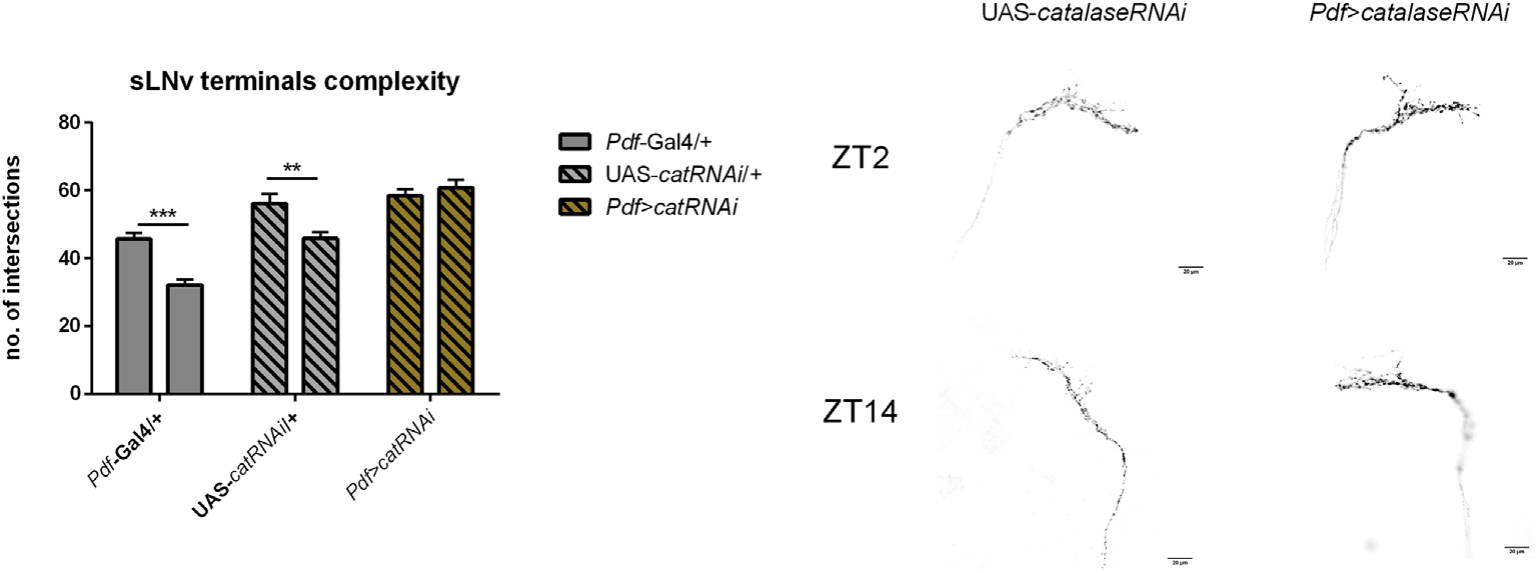
Changes in structural plasticity of sLNv terminals in flies with *catalase* silencing in PDF‐expressing cells (*Pdf>pleRNAi*). Daily remodeling of sLNv processes of the parental flies shows daily changes (*Pdf*‐Gal4 **** *P* = 0.0003, *N* = 32–37, UAS‐*catRNAi* ** *P* = 0.0057, *N* = 32–48) while in *Pdf>pleRNAi* flies it is not maintained (*P* = 0.8111, *N* = 44–54). Bars represent average value ± SEM. Every experiment was repeated three times. Scale bars = 20 μm.

**Fig. 16:**
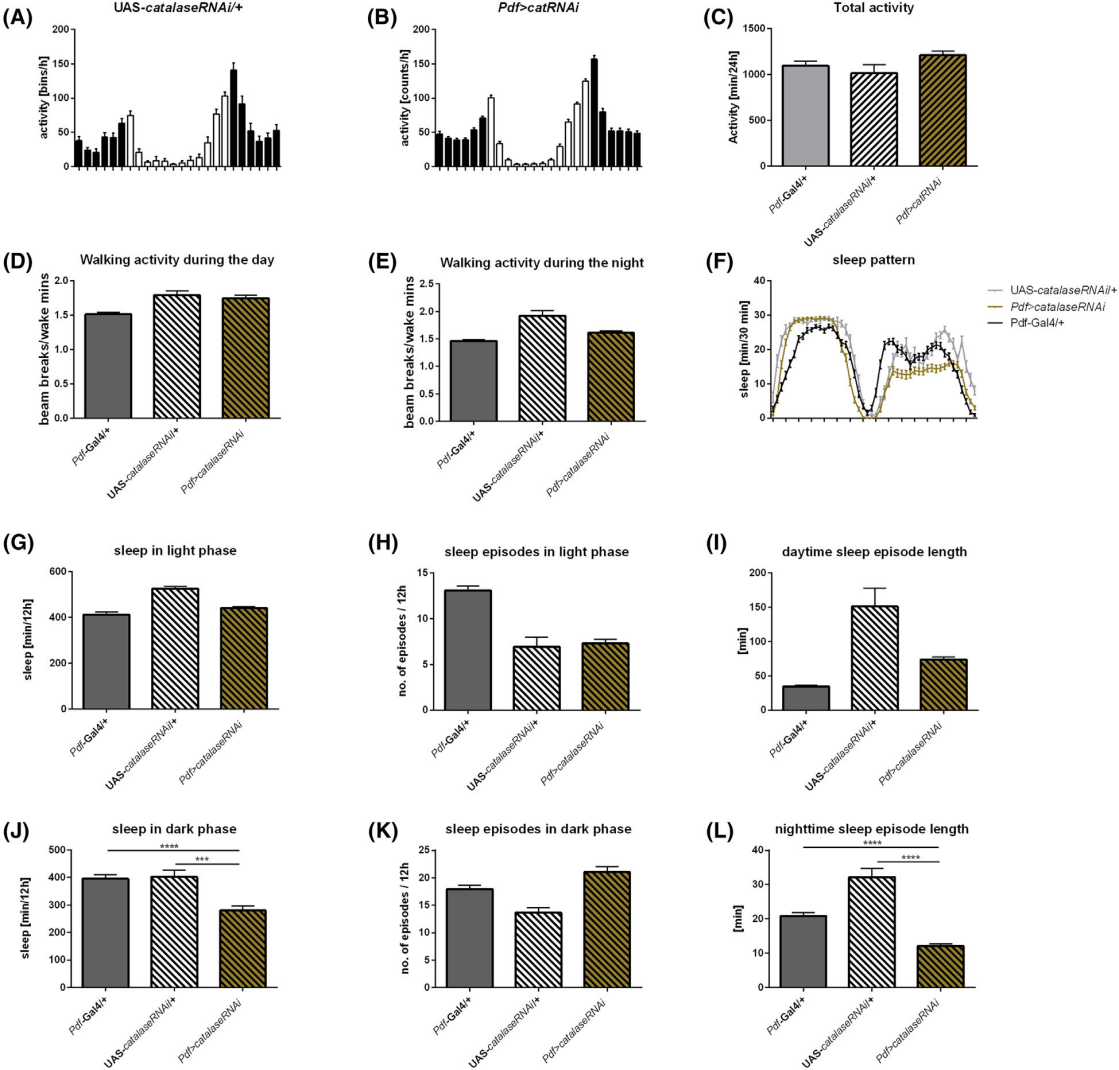
Oxidative stress in clock neurons (*Pdf>catRNAi*) decreases sleep time during the night. (A, B) Daily activity profile of control (UAS*‐catalaseRNAi*) and experimental flies. (C–E) Total activity and walking activity of experimental flies are not affected. (F) Daily sleep pattern shows decreased sleep during the night (ZT15‐ZT21). (G–I) Sleep time, the number of episodes, and bout length during the day are not changed. (J) Sleep time during the night is decreased compared with both controls (Bonferroni's test, **** *P* < 0.0001 with UAS‐*catRNAi*, *** *P* = 0.006 with *Pdf*‐Gal4). (K) The number of sleep episodes during the night is not affected. (L) The length of sleep episode during the night is shorter than that of control flies (Bonferroni's test, **** *P* < 0.0001). The number of flies analyzed: *Pdf*‐Gal4 *N* = 52, UAS‐*catalase N* = 32, *Pdf>catalaseRNAi N* = 83. Bars represent average value ± SEM. Every experiment was repeated three times.

In addition, genetic modification of catalase expression (*Pdf>catRNAi*) in the pacemaker cells, which can inhibit cellular response to ROS, dysregulated neuronal plasticity of sLNv (Fig. 15). However, the effect on sleep was observed only during the night (Fig. 16).

## Discussion

Disruption of the circadian clock is commonly observed in aged people and patients suffering from neurodegenerative diseases such as Parkinson's disease (PD). Although the most severe symptoms are motor ones, connected with dopaminergic neurons loss in the substantia nigra pars compacta, sleep and circadian dysfunctions are very typical for this disease. These kinds of symptoms can be observed years before the onset of motor symptoms and could be an early sign of the disease progression. All studies on PD are still not conclusive whether clock disruption affects the neurodegeneration rate or PD progression enhances the clock disruption. In PD patients, sleep problems affect normal daily functioning and decrease life comfort. Moreover, clock disruption increases the risk of psychiatric disorders, such as depression. The understanding of mechanisms involved in clock changes during PD could provide more effective treatments.

The fruit fly, with its well‐recognized neuron connectome and molecular pathways in clock and sleep neurons, could serve as a model to study this problem in detail. It was already shown that clock disruption increases oxidative stress and affects the lifespan [8, 20, 21]. In addition, *Clk* mutation in the pacemaker cells increases loss of the PPL1 dopaminergic cell cluster, which is responsible for locomotion decline [22]. However, this effect is clock‐independent but PDF‐dependent. The other dopaminergic cell cluster—PAM, which is postsynaptic to LNd cells, shows increased vulnerability to oxidative stress [22].

The circadian network of neurons in the brain is composed of about 150 neurons grouped in several clusters. They have circadian expression of clock genes, and their rhythmic electrophysiological activity and release of neuropeptides regulate daily changes in the brain functioning and rhythmic behavior. Although they play a major role in shaping the rhythmicity of the body, in fact, they are precisely regulated by other cells, like glia and other neurons, including dopaminergic cells. However, among 150 clock neurons, only PDF‐expressing cells, 3 out of 6 LNds, DN2, and 4 out of 15 DN1s are postsynaptic to dopaminergic cells [23]. Moreover, it has been shown that dopamine regulates sleep through DN1p, affecting daytime sleep [23].

We found that PD model flies, with a homozygous *park* mutation, show abnormal daily activity patterns, without the peak of morning activity and the lack of evening anticipation. In wild‐type flies, light‐on causes an immediate locomotion increase because of the startle response, the reaction to a sudden external stimulus. However, in *park* mutants, this reaction after light‐on in the morning was not observed. The startle response is promoted by activation of photoreceptors in the retina (especially R8) and requires histamine [24]. Surprisingly, the startle response experiment performed in the middle of the dark phase showed increased activity in *park* mutants; however, this effect was shorter than in control flies and was stopped right after light‐off. This effect suggests that *park* mutants are able to recognize light input; however, they have a problem maintaining activity without stimulus, which suggests that pro‐arousal neurons, like sLNv, are not working properly. In addition, unchanged *Hdc* gene expression level suggests that histamine production in the photoreceptors is similar to controls; we cannot exclude the possibility that the activity of the Hdc enzyme is changed or the other sources of histamine are affected by the *park* mutation.

Histamine in photoreceptors is released in the active zone of synapses (AZ), and the main scaffolding protein of AZ is Bruchpilot (BRP). In the photoreceptor terminals, BRP is expressed rhythmically with two maxima at the beginning of the day and night [14]. The morning peak is light‐dependent and is not observed in flies kept in constant darkness [14]. We observed changes in the daily pattern of BRP expression in *park* mutants, with higher expression during the day and at the beginning of the night. Surprisingly, BRP expression was not enhanced after light‐on, which indicates no reaction to light input in photoreceptors. In consequence, the photic stimulus is not transmitted to deeper parts of the brain rhythmically. However, we did not observe decreased levels of BRP in the lamina nor in the brain, as was shown for *pink* mutants [9]. In contrast, the BRP immunosignal was stronger, indicating a higher level of this protein in the lamina. This specific pattern may be related to the dysfunction of CRY, which in the control flies forms complexes with BRP after light exposure and is responsible for BRP degradation during the day [25], or with Atg7 to disrupt autophagy [26].

In addition, we also observed changes in the daily pattern of the alpha subunit abundance of the sodium–potassium pump in the epithelial glia (EG) in the lamina. EG surround cartridges in the lamina, and take part in histamine turnover [15] and in neuroplasticity [27]. The alpha subunit of the sodium–potassium pump shows daily changes in the expression levels, which are regulated by the clock and maintained in constant darkness [17]. Here, again, in *park* mutants, we observed a higher level of the α subunit of ATPase during the day, but a low level during the night. Regulation of the daily α‐ATPase expression is very complex. The main clock neurotransmitter PDF, released by LNvs, enhances its expression in the morning, while Ion Transport Peptide (ITP), specific for fifth sLNv and some of LNd, is involved in the process of downregulation in the middle of the day [18]. Cryptochrome plays a key role in the regulation of α‐ATPase expression during the night and just after light‐on, while clock protein oscillations in photoreceptors are involved in the regulation during the night, but in a CRY‐independent manner [18]. It seems that *park* mutants show abnormal activity of glial cells in the visual system. Altogether, the data suggest that both peripheral and central clocks are dysfunctional in the *Drosophila* PD model described in our study.

Visual and circadian signals are integrated by DNp27 neurons, which are more active during the night, and inhibit light‐responsive activity before dawn [28]. Hyperpolarization of these neurons may also be responsible for the lack of the morning peak of activity.

The pro‐arousal clock neurons sLNvs, involved in the regulation of morning activity, also show daily rhythmicity; they change terminal complexity and postsynaptic partners [6, 7]. To check the clock neurons' morphology, we used anti‐PDF immunostaining to visualize pacemaker cells. As a result, we observed abnormal arborization of LNvs in around 12% of *park* mutants (Table 1). This developmental abnormality may explain the greater arrhythmicity of flies noted during the locomotor activity assay (Table 1). Moreover, flies that were rhythmic showed changes in the period of activity rhythm, which corresponds with the previous data [8]. It was previously shown that *per* and *tim* mRNA expression patterns are changed in the head of *park* mutants. In control flies, the maximum of mRNA for *per* and *tim* is observed at the beginning of the night, while in *park* mutants, in the middle of the night, at ZT16, which means it is delayed 4 h [8]. In effect, PER protein level is high during the day, instead of in the late night [8]. In addition, our data showed that the PD model flies do not have oscillations in the sLNv terminal complexity. In wild‐type flies, there are more branches in the dorsal brain in the morning (ZT2) than at the beginning of the night (ZT14) [6]. This pattern was also observed in our control flies. However, *park* mutants show similar complexity of terminals at these two time points. This could be due to disrupted PER rhythmicity, observed in these flies [8]; however, we observed a similar effect after silencing *park* expression in pacemaker cells and cells that are involved in the regulation of sLNv activity, like astrocytes and dopaminergic cells. One of the reasons could be autophagy disruption. PARK protein is involved specifically in the process of mitophagy, but it was previously shown that in the heads of *park* mutants, ATG5 level is also decreased [8], which can affect membrane remodeling necessary for neuronal plasticity. Indeed, autophagy disruption specifically in LNvs affects daily changes of their terminal structures [29], and similar effects were observed after *Atg5* and *Atg7* silencing in astrocytes [30]. Another explanation of this phenomenon could be the decreased expression of *Pdf*, which indeed we observed in *park* mutants. PDF is the main clock neurotransmitter, which is probably produced at a similar level throughout the day, but it is rhythmically released in the dorsal brain [5]. In addition, sLNvs are rhythmically auto‐responsive to PDF [31]. Circadian remodeling of sLNv terminals requires oscillation of PDF release and shows no rhythm and decreased complexity in flies with downregulated PDF levels [32]. This correlates with our data indicating that *park* mutants have decreased *Pdf* expression. In addition, PDF cycling is not required to maintain locomotor rhythmicity in constant darkness [33]. The clock neurons, sLNvs, also use a specific type of autophagy, called secretive autophagy, to release unknown cargo [29]. However, in *park* mutants, although the total level of ATG5 is decreased in the whole head, it is not changed in LNv neurons [8]. Moreover, PARK is linked with dense core vesicles (DCVs) formation, as mutants have a decreased number of DCVs. This phenomenon is observed in the results of increased ER–mitochondria contacts in the cell bodies, which causes pathological changes in lipid composition in the DCV membrane [34]. In effect, PDF accumulates in LNv cell bodies and is not transported to the terminals. It was also shown that an excessive phosphatidylserine transfer at ER–mitochondrial contacts is one of the reasons for sleep changes observed in the PD fly model [34].

In PD, aggregation of alpha synuclein (α‐syn) occurs in the brain. The fruit fly does not express α‐syn; insertion of the human mutated α‐syn gene affects the lifespan and mobility of flies as well as the degeneration of dopaminergic cells, like in PD mammalian models. Alpha synuclein expression, specifically in clock neurons, alters sLNv terminals' structural rhythmicity by affecting lipid metabolism [35]. The level of SREBP, which is a key regulator of lipogenesis, is increased in the brains of flies with expression of mutated SNCA in clock neurons [35]. In the present study, we observed similar changes, with increased *Srebp* mRNA level in *park* mutants. A higher level of *Srebp* expression was also observed in *white* mutants; however, these flies have decreased synthesis of biogenic amines, including dopamine, which indicates that lipogenesis may be important for dopamine metabolism [12]. Moreover, SREBP has been reported as a modulator of the NADP+/NADPH cycle and night time sleep regulator [36].

Analyses of sLNv terminal complexity showed similar changes after mitophagy disruption in cells of the whole organism and in different cell types. In *park* mutants, a higher level of ROS was also observed in the body [37]. We expected to see similar effects after silencing of *park* in selected cell types, suggesting that the sLNv remodeling could be affected by increased oxidative stress not only directly in PDF neurons but also in neighboring cells. To check the effect of ROS level on sLNv plasticity, flies were fed with rotenone and H_2_O_2_, and again, the complexity of sLNv terminals did not show changes between morning and evening time points. This result confirms the link between oxidative stress and daily membrane remodeling. Interestingly, clock disruption increases the vulnerability of dopaminergic cells to oxidative stress and increases the risk of death [22, 38]. Our data suggest that increased ROS levels may affect communication between dopaminergic cells, glia, and pacemaker cells, which in effect change daily physiological rhythms in sLNv. It seems that the neuroplasticity of sLNv neurons is highly susceptible to the imbalance of intra‐ and extracellular oxidative homeostasis. In addition, mitophagy disruption affects normal axon patterning during development, as abnormal arborization was observed in mutants and flies with chronic *parkin* silencing, but it was not observed in flies fed with rotenone or H_2_O_2_ in adult life.

Our data showed severe changes in the daily activity and sleep parameters, such as a lack of evening anticipation in activity, sleep fragmentation, and changes in the daily sleep pattern (Table S2). These symptoms are probably related to electrophysiological abnormalities that have been observed in lLNv of the fly PD model [39]. The lLNv mediates arousal behavior [40], and its hyperpolarization disrupts sleep [41]. In *park* mutants, the firing rate of lLNv showed no daily changes affecting activity and sleep [39].

Interestingly, the increased oxidative stress, in both the whole organism and selected cells, increased sleep time during the night. It confirms the previous results, which showed that ROS are cleared during sleep, and a higher level of ROS induces sleepiness [42].

Notably, the cell‐dependent effect of mitophagy disruption on sleep fragmentation was observed. In *park* mutants, the number of sleep episodes during the day and night was increased, while flies with *park* silencing in clock neurons had fewer episodes of sleep only during the night, and mitophagy downregulation in astrocytes affected sleep quality during the day and night. Finally, manipulation of dopaminergic cells affected sleep integrity during the night. Cell types we selected to check regulate sleep by different pathways, directly, by signaling to sleep centers, and via circadian regulation. Dopaminergic neurons activate wake‐promoting cells in the mushroom bodies and fan‐shaped body [43], but at the same time, the other DA clusters regulate lLNv activity [44]. Astrocytes are known to be homeostatic controllers of sleep bouts, and their activity and metabolism affect the fan‐shaped body neurons to induce a resting state [45] and integrate homeostatic and circadian processes to drive sleep–wake cycles [46]. Moreover, wakefulness promotes oxidation in glial mitochondria, and proteins that control mitochondrial damage are required for normal sleep. Mitochondrial metabolism in glia and neurons is linked with sleep in different ways. Activation of mitochondrial damage‐response proteins, such as the dynamin‐like GTPase (Drp1), is induced by ROS and mediates fission of mitochondria and mitophagy. It was shown that Drp1 activity in neurons is required for glial lipid droplet accumulation during wakefulness, whereas Drp1 in glia is necessary for clearance of lipid droplets during sleep [47]. On the contrary, adult‐induced knockdown of *Drp1* in either neurons or glia results in sleep loss and fragmentation [47]. Glial cells are also more resistant to oxidative stress than neurons [47], especially DA neurons, which can explain different effects on sleep quality after mitophagy disruption in these cell types.

Sleep disruptions observed early during Parkinson's disease have a different origin and are enhanced with time. However, increased ROS production, induced by both endogenous factors, such as *park* mutations, and exogenous toxin exposure, strongly affects clock neurons, which are crucial for sleep regulation. The obtained results provided more information about these processes and raised the question of whether the protection against oxidative stress may help to keep the body's clock functional and, in effect, could improve the quality of sleep while PD progresses.

## Declarations

The authors have no relevant financial or nonfinancial interests to disclose.

## Author contributions

MD contributed to the conceptualization. KZ, KS, and MD contributed to the experimental work. MD contributed to the writing – first draft. MD, EP, KZ, and KS contributed to the contributed to the writing – review and editing.

## Supporting information


**Table S1.** Detailed statistics for Fig. 5 and Fig. 6.
**Table S2.** Summary for sleep analysis. The down arrow indicates that the specific sleep parameter is decreased compared with both controls. The up arrow indicates that the specific sleep parameter is increased compared with both controls. A double‐headed arrow indicates that there is no change in a specific sleep parameter.

## Data Availability

The datasets generated and analyzed during the current study are available in the RODBUK repository https://doi.org/10.57903/UJ/A02RIB.
