# Supplementary material for: PARK(ing) time–How park deficiency affects the biological clock in a Drosophila model of Parkinson's disease
Source: FEBS Lett. 2026 Jun 16;600(14):1992–2011. doi: 10.1002/1873-3468.70389 (PMC13404150; doi:10.1002/1873-3468.70389)
Supplement: Supplementary file 1 — Table S1. Detailed statistics for Fig. 5 and Fig. 6. Table S2. Summary for sleep analysis. The down arrow indicates that the specific sleep parameter is decreased compared with both controls. The up arrow indicates that the specific sleep parameter is increased compared with both controls. A double‐headed arrow indicates that there is no change in a specific sleep parameter. [file FEB2-600-1992-s001.docx]

**Supporting Documents**

Supplementary Table S1

Detailed statistics for Figure 5 and Figure 6.

Statistics for fluorescence intensity of BRP immunostaining (Figure 5):

A: CS - Tukey’s test

|  | ZT4 | ZT13 | ZT16 |
| --- | --- | --- | --- |
| ZT1 | <0.0001 | 0.1504 | <0.0001 |
| ZT4 |  | 0.0031 | 0.9902 |
| ZT13 |  |  | 0.0071 |

B: *w^1118^* Tukey’s test

|  | ZT4 | ZT13 | ZT16 |
| --- | --- | --- | --- |
| ZT1 | 0.0007 | 0.0013 | 0.1426 |
| ZT4 |  | <0.0001 | 0.3146 |
| ZT13 |  |  | <0.0001 |

C*: park* mutant – didn’t pass normality test, nonparametric Dunn’s test

|  | ZT4 | ZT13 | ZT16 |
| --- | --- | --- | --- |
| ZT1 | 0.0042 | 0.0416 | 0.09 |
| ZT4 |  | >0.9999 | <0.0001 |
| ZT13 |  |  | <0.0001 |

Statistics for fluorescence intensity of αATPase immunostaining (Figure 6):

D: CS - Tukey’s test

|  | ZT4 | ZT13 | ZT16 |
| --- | --- | --- | --- |
| ZT1 | <0.0001 | 0.013 | <0.0001 |
| ZT4 |  | <0.0001 | 0.9931 |
| ZT13 |  |  | <0.0001 |

E: *w^1118^* Tukey’s test

|  | ZT4 | ZT13 | ZT16 |
| --- | --- | --- | --- |
| ZT1 | <0.0001 | <0.0001 | <0.0001 |
| ZT4 |  | <0.0001 | 0.9798 |
| ZT13 |  |  | <0.0001 |

F*: park* mutant – didn’t pass normality test, nonparametric Dunn’s test

|  | ZT4 | ZT13 | ZT16 |
| --- | --- | --- | --- |
| ZT1 | >0.9999 | <0.0001 | <0.0001 |
| ZT4 |  | 0.0003 | 0.0004 |
| ZT13 |  |  | >0.9999 |

Supplementary Table S2

The summary for sleep analysis. The down arrow indicates that the specific sleep parameter is decreased compared to both controls. The up arrow indicates that the specific sleep parameter is increased compared to both controls. A double-headed arrow indicates that there is no change in a specific sleep parameter.

|  | Total activity | Walking activity | Sleeptime  DAY | Sleeptime NIGHT | Sleep fragmentation DAY | Sleep fragmentation NIGHT |
| --- | --- | --- | --- | --- | --- | --- |
| *park^1^* | **↓** | **↑** | ↔ | **↑** | **↑** | **↑** |
| *Pdf>parkRNAi* | **↓** | ↔ | ↔ | **↑** | ↔ | **↓** |
| *alrm>parkRNAi* | **↓** | ↔ | ↔ | **↑** | **↑** | **↑** |
| *Ple>parkRNAi* | **↓** | **↓** | ↔ | **↑** | ↔ | **↑** |
| rotenone | ↔ | ↔ | ↔ | **↑** | **↑** | **↓** |
| H_2_0_2_ | **↓** | **↓** | ↔ | **↑** | **↑** | **↓** |
| *PPdf>catalaseRNAi* | ↔ | ↔ | ↔ | ↔ | **↓** | ↔ |
